# Thinking differently: Re‐framing family violence responsiveness in the mental health and addictions health care context

**DOI:** 10.1111/inm.12641

**Published:** 2019-08-23

**Authors:** Jacqueline Short, Fiona Cram, Michael Roguski, Rachel Smith, Jane Koziol‐McLain

**Affiliations:** ^1^ Te Korowai Whāriki, Central Regional Forensic and Rehabilitation Mental Health Service, 3 DHB Mental Health Addictions & Intellectual Disability Service Wellington New Zealand; ^2^ Katoa Ltd Auckland New Zealand; ^3^ Kaitiaki Research and Evaluation Wellington New Zealand; ^4^ Family Violence Death Review Health Quality & Safety Commission Wellington New Zealand; ^5^ Department of Nursing Auckland University of Technology Auckland New Zealand

**Keywords:** intimate partner violence, mental health services, New Zealand, racism, substance addiction services

## Abstract

Aotearoa New Zealand’s high rates of intimate partner violence (IPV) and child abuse and neglect point to a clear need to develop and resource equitable mental health and addiction practices that are responsive both to people experiencing and using violence, and to their families. Current responses to IPV in mental health and addiction settings in Aotearoa New Zealand require a critical re‐framing, from an individualistic autonomy and empowerment framework that constrains practitioners’ practice, to an understanding IPV as a form of social entrapment. Using a composite story constructed from 28 in‐depth New Zealand family violence death reviews, we highlight current problematic practice and discuss alternative responses that could create safer lives for people and families. Re‐framing IPV as a form of social entrapment acknowledges it as a complex social problem that requires collective steps to secure people’s safety and well‐being. Importantly, a social entrapment framework encompasses interpersonal and structural forms of violence, such as the historical and intergenerational trauma of colonization and links to ongoing structural inequities for Māori (the indigenous people of Aotearoa) in Aotearoa New Zealand.

## Introduction and Aims

Violence against women is a significant yet preventable global public health problem (World Health Organization, 2013). As Kaseba‐Sata (2014) states, “Almost all gender‐based violence victims fall on the doorstep of the health sector” because the health sector is the institution that victims are most likely to engage with, most often. Despite this, responding to family violence within health care settings is generally poor, as it is treated and resourced as a marginal health issue (Breckenridge & Salter, 2012). This is particularly problematic in mental health and addiction (MH&A) services due to the significant relationship between mental health disorders and substance misuse for people experiencing and using violence (Breckenridge & Salter, 2012; Trevillion, Corker, Capron, & Oram, 2016).

The cumulative effect of poor health system responses compounds the risk of enduring multiple harms, and creates a systemic “evolving and prolonged failure” (Vincent & Amalberti, 2016) in the care people receive. This pattern of failure is illustrated in June and Peter’s story (Box 1). Their story is a composite story drawing on real cases and actual quotes from service records, reflecting the knowledge gained by the New Zealand Family Violence Death Review Committee (FVDRC) from 28 qualitative in‐depth death reviews conducted between 2011 and 2018. Throughout this paper, snapshots of June, Peter, and their children’s experiences are used to highlight problematic practice and to discuss alternative responses that could create safer lives for all. The focus of this paper is intimate partner violence (IPV) and its entanglement with child abuse and neglect (CAN).

Box 1 **June and Peter**

June first sought assistance at the emergency department (ED) after Peter repeatedly struck her in the head with a plastic vacuum cleaner tube. She was six months pregnant with their first child. Peter did not believe ‘it’ was his.

Over the next 15 years June, Peter and their four children had ongoing contact with health services that spanned almost the entire health care system. However, June and Peter’s most significant and sustained engagement was through mental health and addiction services for their substance use, and June’s overdoses, long‐term depression, and anxiety.

June said that Peter was very jealous and controlling, and this ‘fuelled his violence’. Anything could set him off; he didn’t need a reason to ‘fly off the handle’. June told health services that Peter was just as bad sober but if he was drinking, it was almost guaranteed something was going to happen. If there wasn’t physical violence on a weekly basis, there was emotional and mental abuse from Peter all the time.

When Peter became angry he would smash up the house and throw food everywhere. Once, he burned her entire wardrobe. June consoled herself by drinking. Their children hated watching her drink and knew that there would be consequences.

Over the years, June disclosed concerns for her and their children’s well‐being and safety to health practitioners. Peter also sought help from health practitioners for his ‘violent temper’ and ‘anger issues.’ Despite there being multiple opportunities to respond proactively, health services did not initiate safety strategies that could have contributed to preventing Peter killing June, 15 years after this first ED presentation.

## Background

### The New Zealand Family Violence Death Review Committee

The New Zealand Family Violence Death Review Committee (FVDRC) is one of five mortality review committees hosted by the Health Quality & Safety Commission (the Commission). The Commission assumed responsibility for mortality review in Aotearoa New Zealand following the New Zealand Public Health and Disability Amendment Act 2010. The overarching goal of the FVDRC is to contribute to the prevention of family violence and family violence deaths (Family Violence Death Review Committee Terms of Reference, 2015).

### Death review methodology

The FVDRC has developed a system designed to collect a minimum set of information about all family violence deaths in Aotearoa New Zealand, while selecting some death events for additional in‐depth, multi‐sectoral review. These in‐depth reviews examine qualitative information and narratives in historical and contemporary case files from a range of services, with the purpose of seeing how the family violence system responded to people and their families and whānau (indigenous New Zealand Māori extended family group) involved in the death events. The reviews are undertaken with regional panels, which include representatives from the key agencies involved in the family violence response along with family violence and cultural experts. Review preparation includes: (i) a traumagram to map experiences of trauma for the families and whānau involved (often over four generations), (ii) collation of narrative lifestories of the people involved and (iii) a multi‐chronology timeline of key events, agency practice and collaborative work. Analysis is aligned with understanding response as a complex adaptive system (Braithwaite, Churruca, Long, Ellis, & Herkes, 2018; Davidson‐Knight, Lowe, Brossard & Wilson, 2017). Additional details of the review methodology are available in Committee reports and publications (Family Violence Death Review Committee, 2016, 2017; Tolmie, Wilson, & Smith, 2017).

### The colonization of Aotearoa New Zealand

In Aotearoa New Zealand, to understand the over‐representation of Māori (the indigenous people of Aotearoa) affected by family violence and in the MH&A system (Rangihuna, Kopua, & Tipene‐Leach, 2018), the historical and contemporary consequences of colonization must be acknowledged. Violence against Māori wāhine (women) and mokopuna (children and grandchildren) is not part of traditional Māori culture (Te Puni Kōkiri, 2010). Rather, the level of violence within whānau seen today reflects the patriarchal norms of the British colonizing culture, as well as historical and intergenerational trauma from the widespread and ongoing fragmentation of Māori social structures that were enforced during and after the colonization of Aotearoa New Zealand (Family Violence Death Review Committee, 2017).

Historical and intergenerational trauma links the ongoing structural inequities arising from the large‐scale confiscations and theft of Māori land and other resources by colonizers and the ensuing stigmatization and marginalization of Māori (Reid & Cram, 2005; Wirihana & Smith, 2014) to present‐day trauma‐related physical and mental health outcomes (Walters *et al.,* 2011). Understanding the social, political, and historical contexts impacting Māori is critical in terms of any MH&A practice responses.

### Thinking about intimate partner violence

Health system responses to family violence in Aotearoa New Zealand have traditionally been founded on an empowerment framework that relies on victims who have already sought help from health care services to subsequently be responsible for seeking further help from family violence services (Wilson, Smith, Tolmie, & de Haan, 2015). This response fits with the neoliberal discourse of individualistic self‐management (Beddoe & Keddell, 2016; Woodall, Warwick‐Booth, & Cross, 2012). Such a ‘response’ leaves women and children at risk of further harm by individualizing a complex social problem that requires collective steps to secure people’s safety and restore well‐being (Family Violence Death Review Committee, 2016).

Within MH&A services such ‘responses’ are further conflicted as autonomy is a fundamental principle of ethical medical practice (O’Neill, 2002) and mental health practitioners are rightly mindful of coercive or paternalistic practice. New Zealand mental health legislation (such as the Mental Health (Compulsory Assessment and Treatment) Act 1992), has attracted growing criticism for not reflecting modern approaches to human rights, supported decision‐making and informed consent (Government Inquiry into Mental Health & Addiction, 2018). It is therefore unsurprising to hear mental health practitioners state that while they can give safety advice to victims, it is the victims’ choice about whether or not they take that advice. We suggest that re‐framing IPV as a form of social entrapment is a way of thinking about IPV in MH&A settings which can support different practice responses to those experiencing or using violence (Box 2).

Box 2 **IPV as a form of social entrapment**

IPV is a gendered pattern of harm operating as a form of social entrapment (Ptacek, 1999). There are three dimensions:

1. social isolation, fear, and destruction created by her partner’s coercive controlling behaviours.
2. lack of responsiveness of powerful institutions to her help‐seeking and suffering; and
3. exacerbation of his abuse by structural inequities – colonization, racism, sexism, poverty, heteronormativity, and disability.

This framing of IPV makes visible the partner’s specific pattern of coercive controlling behaviours and how these have limited her ability to be self‐determining (i.e. he attends all health care appointments with her), whilst simultaneously revealing the inadequacy of services’ responses to her help‐seeking (i.e. provision of rote safety advice), and the wider operations of power and privilege in her life (i.e. institutional racism).

Understanding IPV as a form of social entrapment, with individual, collective and structural dimensions, creates several shifts in our thinking (Family Violence Death Review Committee, 2016; Tolmie, Smith, Short, Wilson, & Sach, 2018):

- victims’ responses to abuse are acts of resistance not empowerment. They resist their partner’s abuse, but their resistance does not stop his abuse.
- victims’ help‐seeking is not the problem, but rather the organizational responses to their help‐seeking are.
- we must change the unjust social conditions of people, their whānau or family’s lives, rather than asking them to adjust to oppression.

### Asking about intimate partner violence

The FVDRC in‐depth reviews reveal that victims are proactive help seekers. However, their help‐seeking approaches are often missed by time‐pressured health practitioners who treat the surface issues they are trained to see (Box 3). The reviews provide evidence of an over‐reliance on routinized, rule‐based, ‘tick‐box’ screening to identify victims. Such processes can result in health practitioners not seeing the complexity of what lies behind victims’ presentations, health signs and symptoms (Wilson, 2000), and failing to respond therapeutically (Sampson & Read, 2017).

Box 3 **Seeing but not seeing**

Sixteen weeks pregnant with their third child, June presented again at the ED. She had overdosed on prescription medication. The ED hospital social worker made a report of concern to the statutory child protection agency. The reason for June’s overdose was recorded as ‘social stressors i.e. finance.’ Peter was noted in the report of concern as being fully cooperative and ‘open and honest’ about a pending ‘Male Assaults Female’ court appearance. They were both undertaking relationship counselling for ‘family violence issues.’ Maternal mental health (MMH) (a service for expectant and new mothers) would follow‐up concerns about June’s emotional well‐being and pregnancy status.

The child protection agency visited their home and their two children were ‘sighted’. The agency record stated the concerns had been addressed as the partner was at home at the time of incident. ‘Mother said she lost it with the finances and was very impulsive and foolish. She will talk things through in the future’. The following supports were noted in the agency record as in place for June:

1. Partner support
2. Family Start [home visiting programme that works with vulnerable 0–5‐year‐old children and their families] wraparound services
3. MMH no clear diagnosis at this stage, possible post‐natal depression, on medication, being monitored
4. Relationship counselling
5. Work and Income New Zealand finance‐appointment set for Monday.

‘Children are safe as there is a protective adult (father) in the home as well as extended family.’ Case closed.

MMH followed up with June. They screened her for IPV, but when she did not verbally respond, they did not ask again.

In practice, identifying victims is a “complex process that requires more than asking questions and following the steps of a protocol” (Goicolea, Hurtig, San Sebastian, Vives‐Cases, & Marchal, 2015). Practitioners needed to ask June about her social history and pay close attention to the multiple forms of communication co‐occurring – listening for the feelings behind her words, hearing what she said and did not say, and being aware of her and their own body language (World Health Organization, 2014, p. 17).

Taking a comprehensive history in a MH&A setting involves sensitive inquiry into aspects of people’s lives, the contexts in which those lives are lived, experiences of mental distress and ill‐health, and trauma and violence (interpersonal and structural). Information is gathered about whānau and families to provide a wider context, with the understanding that information is to be respected and safely and confidentially held. Disclosing trauma and violence in a clinically and culturally safe environment, where experiences are acknowledged through a mental health assessment/history‐taking process, is an important first step in creating a practice setting in which a victim can begin to feel respected, supported, and truly listened to.

### Keeping victims safe

A key theme from the in‐depth reviews is the assumption that victims are somehow responsible for managing their own safety (Box 4). The concepts of autonomy and empowerment are particularly problematic when working with victims facing lethal violence, who also frequently face severe structural disadvantages. These concepts can make it appear that a victim’s inability to keep herself or her children safe is a result of her own poor decisions and choices (Wilson *et al.,* 2015).

Box 4 **Safety as an exercise in self‐management**

Late one evening in the year of her death, June left a desperate phone message for a nurse at the Addictions Service that she had worked with in the past. The following day, the nurse called June. June sounded intoxicated and was very tearful as she disclosed years of abuse at Peter’s hands. She said, ‘something has been brewing for a while’. Peter has always had a ‘temper’, but she had noticed an increase in his intimidatory ways – like him pretending to punch her in the face to scare her. Their four children were living with relatives, and her family had ‘wiped their hands’ of her.

The nurse gave June the number of the local family violence service. It was noted that June knew to ring Police ‘but won’t do that’. June guaranteed her own safety, specifically denying thoughts of harming herself or others. As she had returned to daily drinking and was not a current client, she was encouraged to seek treatment for alcohol use. It was noted that she was aware of the Addiction Service contact details.

The next day the nurse called June again. There was no answer. A week later June self‐referred to the Addictions Service. A family violence risk assessment was undertaken with her, and a referral was made to the local family violence service.

Over the next few months June had sporadic contact with the Addictions Service. A cycle began whereby June called to cancel appointments as she was ‘embarrassed’ about her bruises, her body ached, and she had no transport. The Addictions Service called to remind her of appointments. She continued to relay ongoing assaults from Peter and not wanting to call the Police. At one point she was contemplating Women’s Refuge: ‘I am reading their brochure now’.

The Addictions Service was concerned so they called the local family violence service, who had met June once. She was drunk and unable to take their advice. The practitioner said ‘June is an alcoholic. We arranged to meet her when she was sober, however, she did not show. If June does not want our help, there is nothing we can do.’ They advised not texting or visiting as this would make life harder for June. They suggested calling and hanging up if Peter answers.

Reponses focussed on autonomy and empowerment ignore any responsibility of services, including MH&A services, to take safety‐enabling actions that actually help victims. It is clear that June was unable to ‘keep herself safe’ from Peter’s violence, this was why she asked for help. Instead of Peter’s violence being the problem, June became the problem. Her lack of ‘engagement’ with the paucity of what was offered deemed her to be someone who was not wanting help and in effect, ‘choosing’ to be abused.

Health service responses to victims usually involve running through a rote safety checklist (e.g., having a bag packed, money, calling the Police, and accessing Women’s Refuge), without consideration of what victims have already tried, what helped (or did not), how their partner reacted and what actual access to social, economic, and cultural supports they may have. June was never asked why she did not want to call the Police, despite being repeatedly told to do so. What were her fears and previous experiences? Giving advice without first comprehending the unique configurations of entrapment operating in a victim’s life undermines her dignity and is not empowering (Stark, 2012).

To initiate steps to safety, practitioners need to act as safety allies with victims (Family Violence, Sexual Violence & Violence within Whānau: Workforce Capability Framework, 2017). This requires practitioners to ‘create relationships of dignity and respect, to build a bridge to meet the person where they are at’ (Reynolds, 2019, p. XII). From this place, people can make choices where they have actual choices and access to power, but they are not held responsible for another person’s violence, for creating safety for themselves and their children, or for the daily struggle of a life underscored by precarity and marginalization.

### Developing practice with men who use violence

The health system’s blinkered focus on victims has obscured the development of responses to men using violence following disclosures from victims and these men. This misses vital opportunities for health care professionals to help men understand and change their behaviours (Tarzia, Forsdike, Feder, & Hegarty, 2017).

In the in‐depth reviews, men using violence were referred to mental health services because practitioners believed that their abusive behaviour originated in a mental health condition, or because of their suicidal ideation or depression. Other men had self‐referred to community drug and alcohol services or were referred as a condition of a community‐based sentence. Within MH&A services, there are no formal organizational approaches to support practitioners working with men using violence, consequently many of the contacts with these men did not address their use of violence in ways that strengthened safety responses for child and adult victims (Box 5).

Box 5 **Misunderstanding men’s use of violence**

Peter self‐referred to a substance abuse service the month before killing June, after having spent most his life trying to avoid MH&A services. Oranga Tamariki (the New Zealand statutory child protection agency) were now involved with their children. Their involvement and memories of his own childhood had spurred Peter to seek help. He requested entry to a residential programme and was told that it could take some weeks.

The following week, Peter and the counsellor began assessing his suitability for a placement. Peter said that June called him an angry man. He was sure she was planning to leave him as he had found a Women’s Refuge pamphlet hidden at home. He was assessed as dependent on alcohol and methamphetamine. The counsellor noted Peter’s substance abuse gets him down, angry, frustrated, and hating. Withdrawal triggered irritability, low mood, and anger. ‘Sometimes I can snap out of it, other times I can’t,’ he said. The counsellor listed his problems as addiction, coping strategies, and relationship problems.

The counsellor developed a safety plan with Peter until he got a residential placement. Peter was to ‘walk away’ from June, family and friends if he found himself escalating or becoming violent. Both Peter and his counsellor viewed his increased substance abuse as the trigger for his anger and violent temper towards June and others.

Without specific family violence needs assessment and risk management processes informing the care pathways in MH&A services, responses default to individual practitioner’s knowledge and skills. The practitioners involved with Peter minimized his violence and misunderstood it as symptomatic of his alcohol and drug problems, hence missing the potential risk he posed to June and their children.

Peter’s self‐report was the main source of information for the assessment process. Historically, MH&A practitioners may have struggled with disclosure of information for fear of falling foul of privacy legislation. However, the information‐sharing provisions of the New Zealand Family Violence Act 2018 clearly stipulate that family violence agencies (which includes district health boards, New Zealand’s twenty regional health service funding and provision bodies) may request personal information about a victim or person using violence for the purposes of a family violence risk or need assessment, to contribute to a response to family violence, and/or to protect a victim from family violence (Family Violence Act 2018). With respect to family violence perpetration, MH&A assessment processes need to seek third‐party information and to corroborate self‐reported information with other services, such as the statutory child protection agency, the Police and local support services.

For Peter, the suggested intervention for when he was feeling angry was to remove himself from the situation. This was Peter’s responsibility, with no additional agency support. To provide victims with safety, and support realistic behaviour changes for men using violence, MH&A services need to work in partnership with specialist family violence services and contribute to local family violence multi‐agency review processes for individuals’, whānau and family’s safety and well‐being strategies.

Promoting and respecting men’s capacity to change and take responsibility for their actions needs to sit with all practitioners and organizations. Rather than working in isolation or ‘signing off’ after referral, MH&A services require capability support to partner with statutory and specialist community services working with men using violence (Victoria State Government, 2017). This requires developing reciprocal relationships with local family violence multi‐agency response initiatives, as they are often working with the same people. For example, a 12‐week review of 129 Integrated Safety Response (a government family violence multi‐agency pilot involving health services) cases identified drug and alcohol addiction as a risk factor for 60 percent of people using and experiencing violence. Mental health support was an identified need for 21 percent of victims and 41 percent of people using violence (Ryan & Block, 2019).

In the following box are reflective questions about IPV responsiveness for MH&A practitioners.

### Ensuring equitable health care practice

#### Preventable health burdens

There is an ever‐increasing evidence base of the short‐ and long‐term physical, mental, sexual, and reproductive health outcomes from experiencing family violence across one’s life span (Webster, 2016). Comparatively, far less attention has been directed at understanding how people’s individual and collective experiential ‘health burdens’ are shaped and compounded by poor organizational responses and the embeddedness of structural inequities. Such an approach seeks to question and address why victims who receive *negative* responses to their help‐seeking are more likely to receive a diagnosis of a mental health disorder (Richardson & Wade, 2010); why women with pre‐existing depression or major mental health disorders are more vulnerable to experiencing IPV victimization and re‐victimization (Khalifeh *et al.,* 2015; Trevillion *et al.,* 2016); and why Māori whānau seeking help continue to experience institutional racism (Dhunna, Lawton, & Cram, 2018; Government Inquiry into Mental Health & Addiction, 2018; Inquiry into Mental Health & Addiction, 2019; Wilson & Webber, 2014).

Evidence from the indepth reviews indicates that if June or Peter were Māori and of low socio‐economic status, they would be more likely to experience stigmatization, racist attitudes, and indifference to their suffering. These responses would occur in wider society, in their access to the resources of that society and the determinants of health, and when they sought help from MH&A services as well as other agencies (Box 6 and 7) (Family Violence Death Review Committee, 2017).

Box 6 **Questions for mental health and addictions practitioners**

- Have I assessed the risks of her partner’s coercive and controlling behaviours towards her and the children?
- How do his coercive and controlling behaviours constrict her and her children’s lives and her ability to do what she wants to do, including her ability to formulate and engage in any MH&A care plans?
- What do I know about what safety strategies she previously tried, how these worked, if services were helpful, her partner’s reactions, and what if any access she has to financial, family and whānau, social and cultural supports?
- Are she and her family and whānau experiencing systemic barriers, such as a lack of stable housing, limited access to money and transport, poverty, and dismissive racist responses from services? How is this impacting her, her children and whānau and family’s safety and wellbeing?
- What are her biggest fears for her and her children?
- Who is working with her partner? What strategies are in place to support him and address his use of violence?
- Comprehending all of this, what actions can I take as a ‘safety ally’, as part of my treatment plan?
- How and with who will I review whether what we are doing is supporting creating safety for her, the children and her family and whānau?
- What local Māori and Tauiwi (New Zealand non‐Māori) family violence organizations and networks could we develop relationships, and work in partnership with?

Box 7 **The normalization of racism**

As a child, the Department of Social Welfare (DSW) removed June from her whānau and placed her in a family home run by a Pākehā (a white New Zealander) couple, Fred and Ethel. Her brothers were sent to a borstal. The DSW said that the children were unwashed, had ill‐fitting, and dirty clothing, and were living in deprived conditions. Agencies had tried to work with their parents, but they were found not fit to have care and control.

For years, Fred sexually abused June and the other girls in the home, who were mostly Māori. The DSW seldom visited, and June’s connection to her whānau and whakapapa (Māori lineage) were totally disregarded. Her DSW record stated that she was an underachiever, had poor social skills, and was a ‘candidate for early pregnancy.’ Her brothers, in order to survive in the chronically abusive conditions of the borstal, joined a gang for a much‐needed sense of belonging and self‐protection.

Decades later, a kaimanaaki (Māori person in a role to protect and support) from a mental health service undertook an assessment with June. With respect to her Te Taha Wairua (spiritual well‐being) June said, ‘I feel confused due to the things which have happened to me. I think my wairua (spirit) has been snapped.’

The proposed community mental health services (CMHS) management plan did not include any safety, cultural, or restorative responses. The focus was on harm minimization (to self) and medication. It was assumed by those involved that her life was always going to be ‘gangs, drugs, and getting the bash’.

June did not accept that this was her life; she had repeatedly sought help. She did not feel taken ‘seriously’ by the CMHS. ‘They were only judging me from the way I looked. I really needed help in my head. They weren't being good people.’

The in‐depth reviews, as demonstrated by June’s experiences above, highlight the prevalence of histories of intergenerational trauma and violence, and childhood abuse (including abuse in state care). In the absence of supportive social responses, it was not uncommon for these women and men to self‐medicate with drugs and alcohol as a way of coping with trauma and numbing experiences of abuse. Their experiences echo what many people told the New Zealand Government’s 2018 Inquiry into Mental Health and Addiction. Experiencing CAN was the origin of many people’s mental distress and the ‘trigger’ for ‘counterproductive coping mechanisms such as addiction’ (Government Inquiry into Mental Health & Addiction, 2018). The Inquiry emphasized the importance of trauma‐informed approaches, which do not ask ‘what is wrong’ but rather, ‘what has happened to you?’ (Hopper, Bassuk, & Olivet, 2010; Substance Abuse & Mental Health Services Administration, 2014; Te Pou o te Whakaaro Nu, 2018). However, trauma can also result from ‘what did not happen for you’, for example, when MH&A systems fail to respond to family violence and structural inequities and victims suffer additional harmful health burdens and social consequences (Varcoe, Wathen, Ford‐Gilboe, Smye, & Browne, 2016).

For Māori, an ‘equity’ approach is sourced from the guarantee of citizenship made in the Treaty of Waitangi, Aotearoa’s founding document signed by the Crown and Māori leaders in 1840. Contemporary Māori leaders state the MH&A system is underpinned by ‘unacknowledged institutional racism and indigenous injustices’ and it is ‘in a state of emergency for tangata whenua [people of the land, or Māori]’ (Tumu Whakarae (National Reference Group of Māori Health Strategy Managers within District Health Boards), 2018). This non‐fulfilment of the Treaty of Waitangi guarantee puts the onus on health organizations to remove barriers to Māori being able to journey well to and through health care.

Saliently, Indigenous concepts of health are holistic and ‘constitute more than physical and mental well‐being, or being free from diseases. They incorporate a state of balance between mind, body, and spirit, and of being in harmony with nature’ (International Labour Office, 2003, p. 58). When the Health Practitioners Competence Assurance Act 2004 calls for clinically and culturally competent practice, it is talking about the understanding of a Māori worldview that infuses and tailors health service delivery so that it is responsive to Māori.

### Changing the health care setting not the individual

To address institutional racism and the on‐going impacts of colonization requires changing MH&A settings and the privileging of Western knowledge production systems that underpin practice responses (Borell, Barnes, & McCreanor, 2018), including a shift from trauma‐informed, to trauma‐ and violence‐informed (TVI) practice. Importantly, TVI approaches bring an explicit focus to: *structural inequities* to avoid seeing trauma as happening only ‘in people’s minds’ but also in their social context; *ongoing violence,* as for many people violence is intergenerational and connected to the violence of colonization; and the *responsibility of organizations* to change as systems perpetuate harm (i.e. institutional racism) (Came, 2014; Varcoe *et al.,* 2016).

In Aotearoa New Zealand the development of TVI approaches must be informed by Māori‐specific approaches that stem from the distinctive Māori and Indigenous collective experiences of historical and intergenerational trauma (Cavino, 2016; McClintock, Haereroa, Brown, & Baker, 2018; Pihama *et al.,* 2017). Furthermore, to address those most adversely affected by colonial gender violence and trauma, a mana wāhine (authority, dignity, and power of Māori women) (Simmonds, 2011) historical TVI approach is vital (Cavino, 2019). People do not live single‐issue lives, so we cannot have single‐issue understandings or responses. Many social justice problems like racism and sexism overlap, creating multiple levels of social injustice.

Reflective questions to promote equitable MH&A practitioner and organization responsiveness to IPV are suggested in Box 8.

Box 8 **Questions for mental health and addictions practitioners**

- What kinds of power and privilege do I have? How do these shape my life and world view?
- What kinds of power and privilege do we hold as an organization? How can we use our privileges and power for social justice?
- Have I considered how experiencing trauma and violence may have contributed to the development of the presenting complaint/reason for referral?
- Am I using a culturally responsive, trauma and violence‐informed practice approach to address the presenting complaint/reason for referral?
- How do I support the provision of health care to Māori to be culturally responsive, including respect for and application of te reo (language) and tikanga (protocols) (Ministry of Health, 2014)?
- Do I use assessment tools such as the Meihana Model to infuse culture into clinical assessments that provide a broad picture of patients’ health and well‐being (Pitama, Huria, & Lacey, 2014)?
- What do the patterns of patients’ journeys tell us about the level of responsiveness (including cultural) our organization provides to MH&A patients who are experiencing IPV?
- How does our organization monitor equity and other health improvement targets to ensure that whānau Māori who are experiencing family violence have access to culturally responsive MH&A services (Ministry of Health, 2014)?

### Limitations

The FVDRC has confirmed that the risk of death from family violence is gender‐based, with women and their children as the primary victims and men the predominant aggressors. The FVDRC is aware of the potentially heteronormative focus of its work and is vigilant about the imposition of a classification system that may obscure family violence deaths in same sex and non‐cisgender relationships.

## Conclusions

Current health system responses to family violence are modelled on addressing a simple problem, rather than a complex social problem (Rees & Silove, 2014) that requires a comprehensive and equitable health system response. June and Peter’s composite story illustrates how health system responses to IPV that only focus on empowering victims (including cognitive behavioural therapy, counselling to improve self‐efficacy and self‐esteem, and safety advice), are ineffective at addressing the serious safety issues, structural inequities, and experiential health burdens faced by many victims (Jewkes, 2013; Wilson *et al.,* 2015). This is not surprising. If MH&A services do not partner with specialist family violence services to address men’s use of violence and the ongoing safety and well‐being needs of victims, their families and whānau, then the violence will not stop, and the harm will continue. For Māori this includes the harm through the imposition of Western knowledge production and treatment paradigms. ‘Te Tiriti o Waitangi [the Treaty of Waitangi] must be at the heart of all solutions’ relating to MH&A (Inquiry into Mental Health & Addiction, 2019, p. 3).

## Relevance for Clinical Practice

Health systems need to change to meet people where they are at. The questions in this paper for MH&A practitioners and organizations provide the opportunity to reflect on how understandings of IPV shape practice responses, and how ‘helpful’ these responses are. Acknowledging and addressing the multiple forms of violence and structural inequities many people and their whānau and families experience daily, will require systemic trauma‐ and violence‐informed practice approaches that are truly culturally and collectively responsive (Cram, 2019; Inquiry into Mental Health & Addiction, 2019), holistic and integrated.
